# Melanocyte stem cells in the skin: Origin, biological characteristics, homeostatic maintenance and therapeutic potential

**DOI:** 10.1002/ctm2.1720

**Published:** 2024-05-22

**Authors:** Luling Huang, Yuzhi Zuo, Shuli Li, Chunying Li

**Affiliations:** ^1^ Department of Dermatology Xijing Hospital Fourth Military Medical University Xi'an China; ^2^ Department of Plastic and Burns Surgery The Affiliated Hospital of Southwest Medical University Luzhou China

**Keywords:** hair follicle, melanocyte replenishment, melanocyte stem cells, niche, regenerative medicine

## Abstract

Melanocyte stem cells (MSCs), melanocyte lineage‐specific skin stem cells derived from the neural crest, are observed in the mammalian hair follicle, the epidermis or the sweat gland. MSCs differentiate into mature melanin‐producing melanocytes, which confer skin and hair pigmentation and uphold vital skin functions. In controlling and coordinating the homeostasis, repair and regeneration of skin tissue, MSCs play a vital role. Decreased numbers or impaired functions of MSCs are closely associated with the development and therapy of many skin conditions, such as hair graying, vitiligo, wound healing and melanoma. With the advancement of stem cell technology, the relevant features of MSCs have been further elaborated. In this review, we provide an exhaustive overview of cutaneous MSCs and highlight the latest advances in MSC research. A better understanding of the biological characteristics and micro‐environmental regulatory mechanisms of MSCs will help to improve clinical applications in regenerative medicine, skin pigmentation disorders and cancer therapy.

**Key points:**

1. This review provides a concise summary of the origin, biological characteristics, homeostatic maintenance and therapeutic potential of cutaneous MSCs.
2. The role and potential application value of MSCs in skin pigmentation disorders are discussed.
3. The significance of single‐cell RNA sequencing, CRISPR‐Cas9 technology and practical models in MSCs research is highlighted.

## Graphical abstract

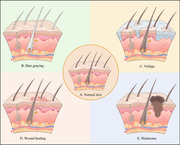

## 1 INTRODUCTION

Melanocyte stem cells (MSCs) are skin stem cells derived from vertebrate neural crests.^1^ During embryonic development, neural crest cells cross the pathway between the somite and the non‐neural ectoderm (dorsolateral pathway) to differentiate into melanoblasts.^2^ Melanoblasts then undergo an extraordinary migration and eventually populate the epidermis and the developing hair follicle (HF).^2^ In the basal layer of the epidermis, melanoblasts differentiate into melanocyte (MC) precursor cells, which are intermediate‐state cells that have not fully developed into functional MCs. In the HF, melanoblasts differentiate into MC precursor cells in the hair bulb and MSCs in the bulge and hair germ (HG) area.^3^ MSCs are located in a microenvironment known as a ‘niche’ with the bulge, which contains multiple stem cells and supporting cells that provide signalling and scaffolding to stem cells.^4^ Both MC precursor cells and MSCs can differentiate into mature MCs that produce melanin, which are essential for maintaining hair and skin pigmentation and vital skin functions.^5^

With the identification of specific lineage markers and advances in stem cell technology, the role of MSCs in the skin is gradually being revealed. The homeostatic maintenance of MSCs is critical for coordinating skin homeostasis, repair and regeneration. More notably, MSCs are closely linked to several skin disorders, including hair graying, vitiligo, wound healing and melanoma. Recognising and understanding the unique biological properties and stem cell–niche interactions of MSCs and their role in skin disease development will pave the way for innovative treatments for a variety of clinical conditions. Unfortunately, there is no available review to refresh the latest advances in MSCs.

Therefore, this review summarises and updates the latest research progress on MSCs, reviews the niche signals that regulate MSCs homeostasis in the HF, and, crucially, discusses the potential therapeutic applications of MSCs in skin pigmentation disorders. The review offers us fresh insights into regenerative medicine, skin pigmentation diseases and skin cancer therapy.

## 2 OVERVIEW OF CUTANEOUS MSCS

### 2.1 Development and localisation of melanocytic lineage

The melanocytic lineage in vertebrates arises from the trunk neural crest.^1^ Between embryonic day 8.5 (E8.5) and E10, transient expression of *Kit* ligands induces neural crest cells’ migration through the dorsolateral pathway to generate melanoblasts. Subsequently, melanoblasts enter the dermis. At around E13.5, they transmigrate from the developing dermis to the epidermis. From E14.5 onward, melanoblasts migrate from the basal layer of the epidermis towards the developing HF, eventually colonising the epidermis and HF and forming different spatially distributed populations, such as MSCs, follicular MCs and epidermal MCs.^2^, ^6^, ^7^ In fact, MCs have a dual neural crest origin and, in addition to the above‐mentioned source, may also originate from Schwann cell precursors, which exploit the ventral migration pathway.^8^, ^9^

**FIGURE 1:**
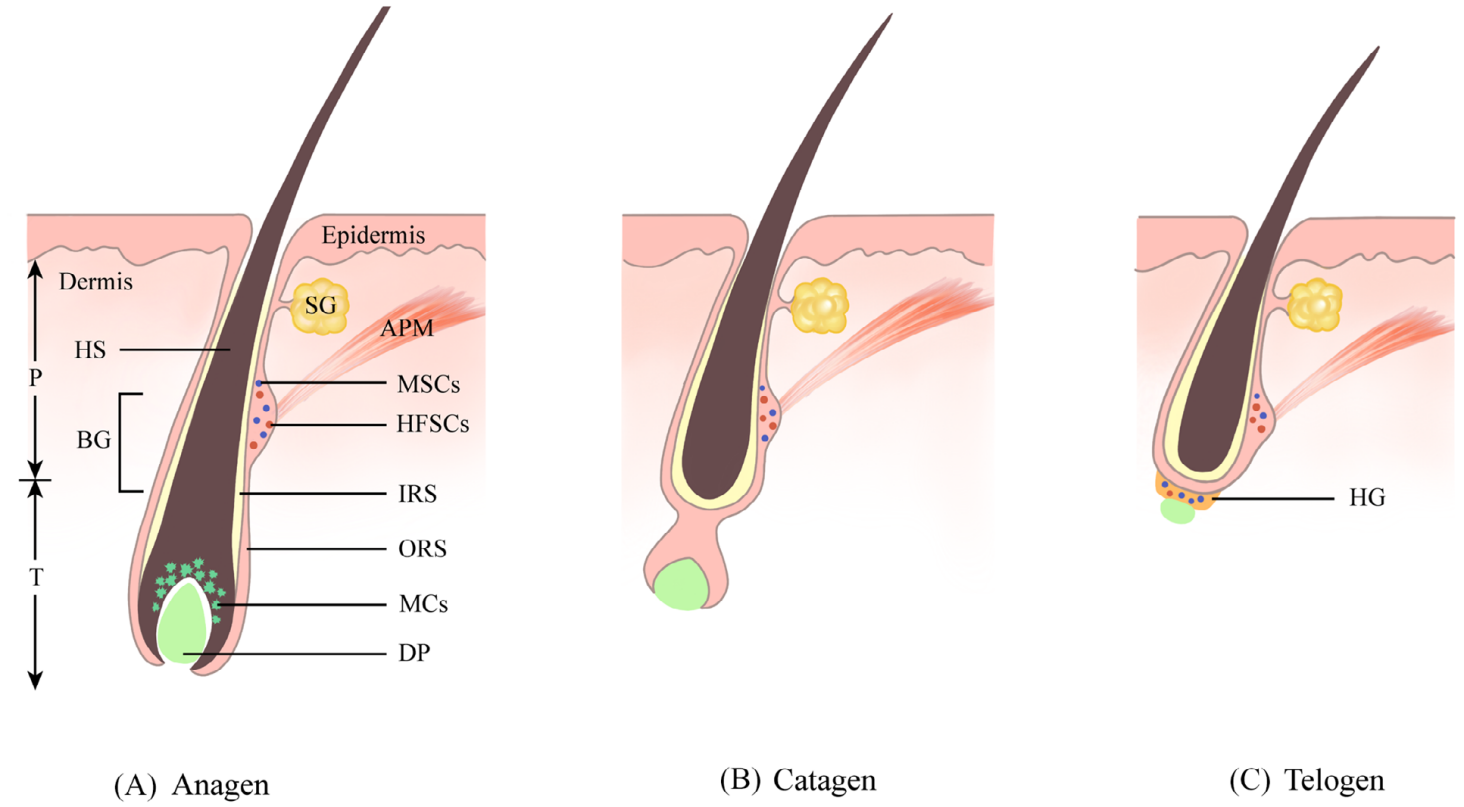
A schematic drawing of the hair follicle structure and melanocyte stem cells (MSCs) and their progeny in the hair cycle. (A) During the early anagen phase, quiescent MSCs (shown as blue dots) positioned in the follicular bulge are activated, migrate down along the outer root sheath (ORS), and differentiate into mature melanocytes (MCs) (shown in green) in the follicular bulb. (B) At catagen, mature MCs undergo apoptosis along with the follicular transient portion, while quiescent MSCs remain in the bulge. (C) During telogen, MSCs maintain a quiescent state in the bulge and hair germ (HG) until the onset of the next anagen phase. APM, arrector pili muscle; BG, bulge; DP, dermal papilla; HFSCs, hair follicle stem cells; HS, hair shaft; IRS, inner root sheath; P, permanent portion; SG, sebaceous gland; T, transient portion.

The HF is a functional appendage of the skin that controls hair growth.^10^ The HF is divided into two compartments: the permanent portion and the transient portion. The permanent portion, the upper part of the HF, includes the infundibulum and the isthmus. The outer hair root sheath (ORS) at the end of the isthmus protrudes outward to form the follicular bulge, which is believed to be the main habitat of hair follicle stem cells (HFSCs).^11^ The permanent portion remains stable and generally does not undergo apoptosis or regeneration during HF morphogenesis. The transient portion, the lower part of the HF, shows morphological changes in growth, recession and quiescence during the hair cycle (Figure 1).^12^ In synchrony with the hair cycle, MSCs in the HF undergo cyclic activation, regression and quiescence phases. During the anagen, quiescent MSCs are activated and migrate from the bulge down to the HF bulb to differentiate into mature MCs. At catagen, differentiated MCs are depleted by apoptosis, although quiescent MSCs remain within the bulge. Upon entry into telogen, MSCs stay stationary in the bulge and HG region (Figure 1).^13^, ^14^ Distinguishing from previous studies that suggested that MSCs are mostly distributed in the bulge, Sun et al. found that during the telogen, MSCs are mainly distributed in the HG region and a few in the follicular bulge (Figure 1).^3^ During the early growth phase, MSCs in the HG are activated and differentiate into transit‐amplifying cells with migratory capacity in the intermediate differentiation state. Transit‐amplifying cells partly migrate to the follicular bulb to differentiate into mature MCs and partly migrate upward to the bulge and exhibit self‐renewal capacity. Due to the lack of differentiation signals within the bulge, the majority of transit‐amplifying cells can de‐differentiate to revert to the stem cell state and return to the HG through the subsequent telogen, thus being used in the next hair cycle.^3^ This pioneering finding provides important proof of the plasticity of MSCs in physiological conditions.

**FIGURE 2:**
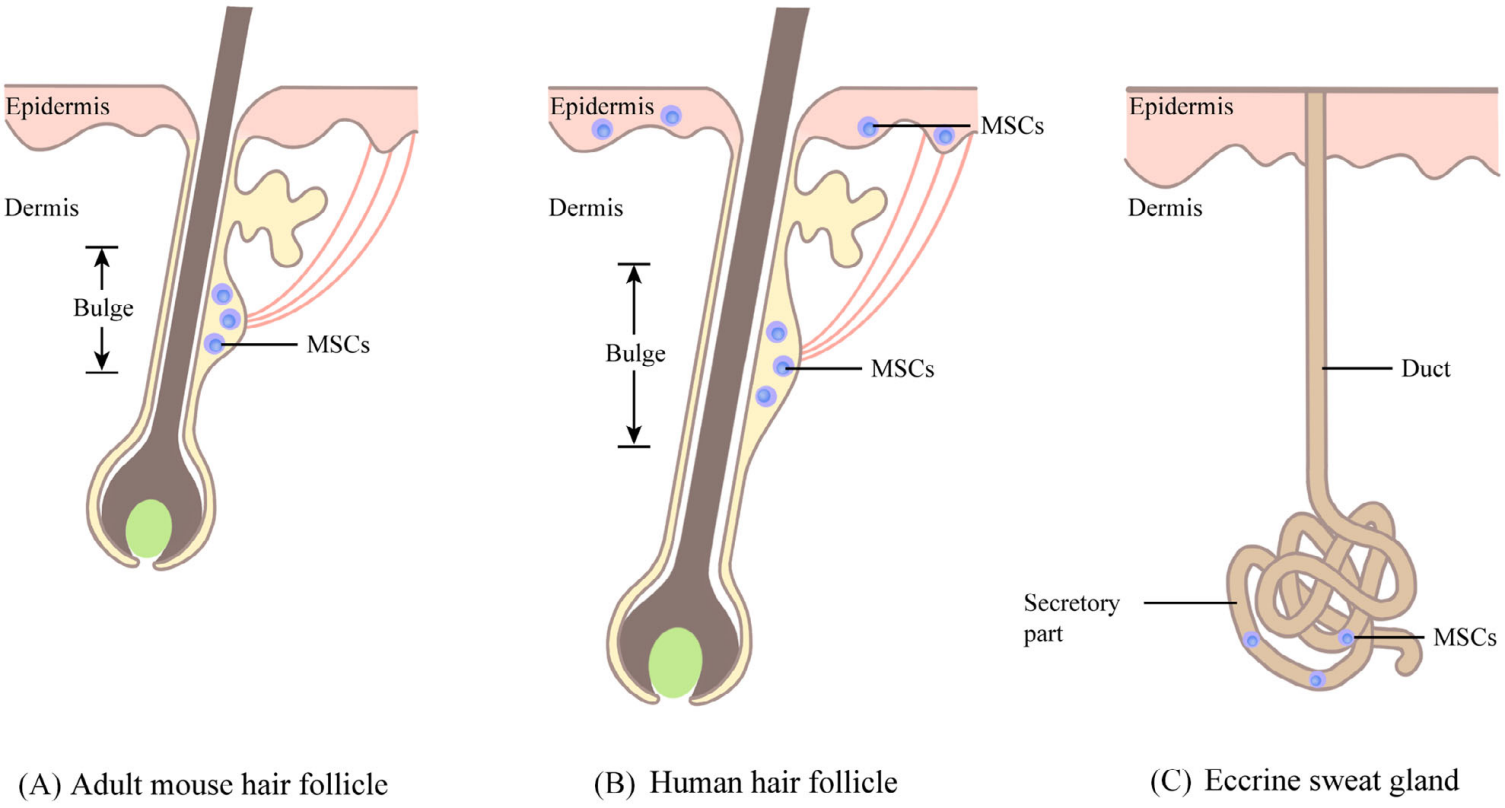
The distribution area of melanocyte stem cells (MSCs). (A) In the adult mouse hair follicle, MSCs are located in the bulge region. (B) In the human hair follicle, MSCs can be identified not only in the bulge region but also on the basement membrane of the interfollicular epidermis. (C) MSCs are present in the secretory part of the exocrine sweat glands in humans and mice.

Interestingly, in adult mouse skin, MSCs are detected in the HF, while in humans, MSCs are also present on the epidermal basement membrane (Figure 2).^15^, ^16^ This might be due to selective expression in different species during the evolutionary process. In addition to HF and the epidermal basement membrane, MSCs have been identified in human and mouse exocrine sweat glands in recent years (Figure 2).^17^, ^18^ Moreover, it has been reported that human subcutaneous adipose tissue harbours MC progenitor cells, which may have the potential to differentiate into mature MCs.^19^ In the future, further studies are necessary to prove whether MSCs exist in them. In conclusion, these MSCs outside the HF not only offer direct proof for the occurrence of MSCs in the extrafollicular dermis but also provide pigment cell sources for the repigmentation of depigmented diseases in certain hairless areas.

### 2.2 Biological characteristics of MSCs

MSCs are small, oval‐shaped cells that have a limited rate of proliferation and lack melanin granules.^20^ MSCs have unique molecular characteristics, and low expression of housekeeping genes and pigment‐related genes is one of the notable features of MSCs. The reported markers of MSCs are summarised in Table 1, including *DCT*,^21^, ^22^ *PAX3*,^23^, ^24^ *MITF*,^24^ *SOX10*,^21^ *c‐Kit*,^25^, ^26^ *PMEL17*,^25^ *FRIZZLED4*,^27^ *FRIZZLED7*,^27^ *CD34* ^28^ and *TFAP2B*.^29^ This significant discrepancy in gene expression strongly demonstrates that MSCs are in an immature state. The 5‐bromo‐2‐deoxyuridine tracking experiment further confirmed that MSCs at the bulge are undifferentiated, cells that are only activated during early anagen, have the capacity to self‐renew, and provide differentiated MCs to the HF.^30^

**TABLE 1:** Reported markers of melanocyte stem cells (MSCs).

Marker	Species	Functions	Ref.
DCT	Mus musculus, Homo sapiens	Promotion of melanin synthesis	^21^, ^22^
PAX3	Mus musculus, Homo sapiens	Maintenance of MSCs quiescence	^23^, ^24^
MITF	Homo sapiens	Regulation of MSCs proliferation and differentiation	^24^
SOX10	Mus musculus	Maintenance of MSCs and regulation of MSCs' fate determination	^21^
c‐Kit	Mus musculus, Homo sapiens	Regulation of MSCs proliferation, migration, and survival	^25^, ^26^
PMEL17	Mus musculus, Homo sapiens	Promotion of melanin synthesis	^23^, ^25^
FRIZZLED4	Mus musculus, Homo sapiens	The receptor for the Wnt signalling pathway	^27^
FRIZZLED7	Mus musculus	The receptor for the Wnt signalling pathway	^27^
CD34	Mus musculus	Maintenance of MSCs identity and multidirectional differentiation potential	^28^
TFAP2B	Danio rerio	Required molecule for melanocyte regeneration from MSCs	^29^

Joshi et al. identified follicular MSCs in the telogen into two subpopulations with distinct functional and regenerative properties for the first time by using *CD34*.^28^ The subpopulation located in the bulge is predominantly *CD34* ^+^‐MSCs with characteristics consistent with neural crest stem cells, expressing high levels of *NGFR*; in comparison to *CD34* ^+^‐MSCs in the bulge, the *CD34* ^‒^‐MSCs subpopulation located in HG is more dominant in MCs differentiation gene expression levels and could induce MCs replenishment more efficiently.^31^ This fact indicates that MSCs still retain their inherent flexibility or sensitivity to cellular reprogramming. The functional heterogeneity of subpopulations of MSCs makes the study of MC differentiation more challenging.

Unlike other adult stem cells, which usually exhibit unidirectional differentiation, MSCs can transform between stem cells and transit‐amplifying cells, which is characterised by ‘reversibility’.^3^ When the MSCs return to HG after completing the HF growth process, they can de‐differentiate to return to the stem cell state, waiting for the next hair growth cycle.

Furthermore, another noteworthy property of MSCs, especially when compared to other stem cell systems, is their isolation. The majority of the time, an HF only houses one MSC, and even when it does, each MSC exists independently of the others.^30^, ^32^ In fact, there are only a few insights into the molecular characteristics of MSCs, especially for human MSCs, due to their scarcity and the difficulty of sample collection. This generates uncertainty in clinical practice and limits our understanding of pigmentary diseases.

### 2.3 Functions of MSCs

MSCs are the cornerstone for maintaining hair and skin pigmentation as well as vital skin functions. MSCs eventually differentiate into mature MCs in the hair cycle that produce melanin and supply it to the hair shaft and are considered to function as a reservoir of MCs for hair pigmentary units.^15^, ^25^ Furthermore, MSCs are a major source of epidermal MCs. UV irradiation or *Kit* ligand expression stimulates MSCs from HF bulge to migrate upward along the ORS to the epidermis and differentiate into mature MCs in the mouse.^25^, ^33^ In addition to being responsible for pigmenting the hair and skin in physiological conditions, MSCs are also involved in repigmentation after various stresses and injuries. These properties of MSCs may be beneficial in the clinical treatment of depigmentation disorders such as vitiligo, a depigmented skin disorder caused by the absence or dysfunction of epidermal MCs.^34^ MSCs in the HF in vitiligo are activated by UV irradiation or drugs, migrate to the epidermis, proliferate and differentiate into epidermal MCs, which secrete melanin and promote repigmentation of the white macules.^25^, ^35^ This pattern of repigmentation, which starts at the orifices of the HF, is called the ‘perifollicular repigmentation pattern’.^36^ Subsequently, the discovery of the ‘medium‐sized spot repigmentation pattern’ indicates that MSCs in the dermis and exocrine sweat glands can also develop into epidermal MCs and participate in vitiligo repigmentation.^37^, ^38^ The various repigmentation patterns that occur are mainly determined by the different sources of residual melanocytic precursor cells, or MSCs, in the epidermis or HF. Obviously, the repigmentation of vitiligo provides an excellent model for investigating MSCs differentiation and function.

The above studies support the notion that MSCs can provide differentiated MCs for hair and epidermis under various physiological and pathological conditions and are crucial for preserving skin function.

## 3 REGULATORY MECHANISMS FOR MAINTAINING MSCS HOMEOSTASIS IN THE FOLLICULAR BULGE

**FIGURE 3:**
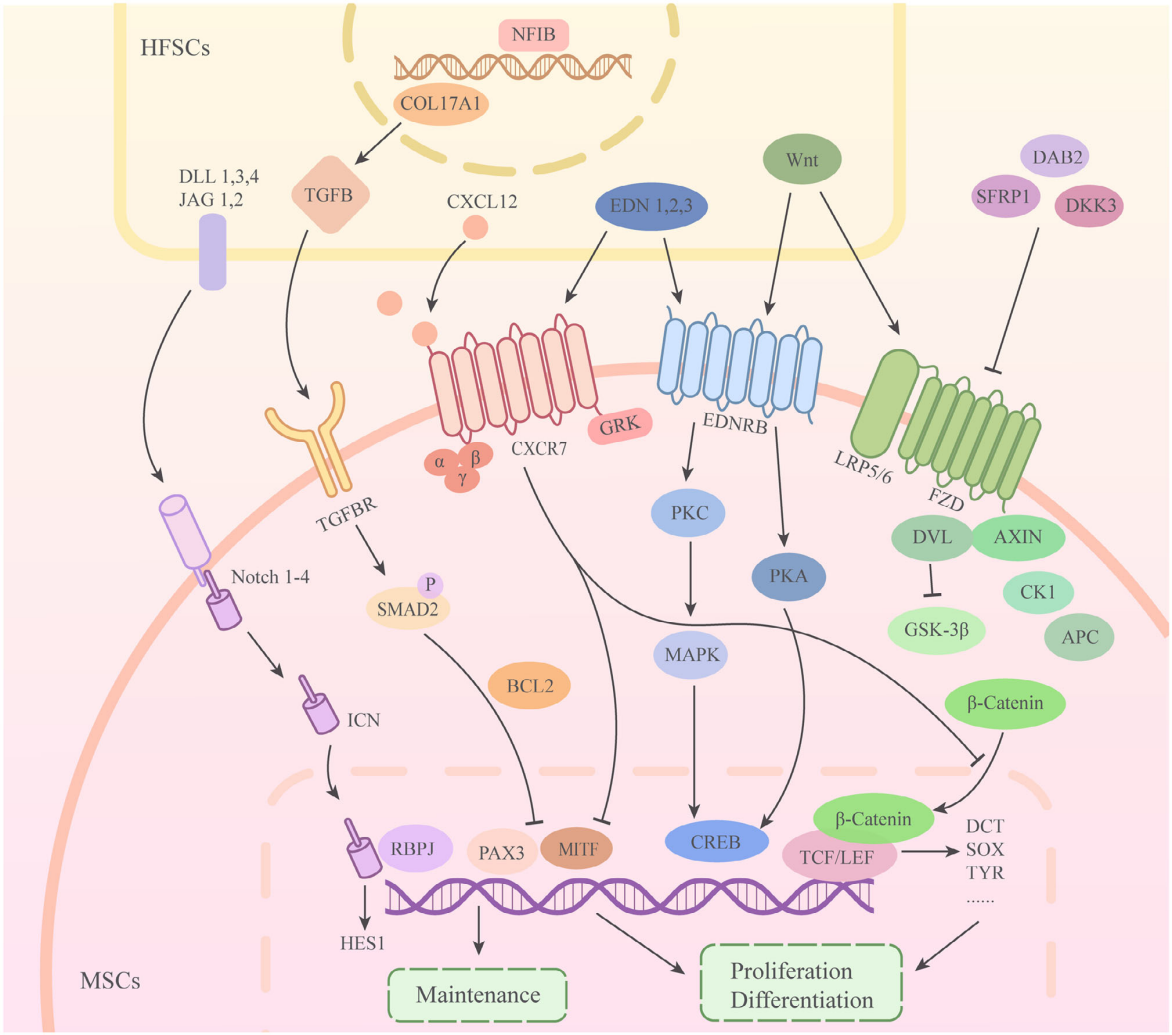
Schematic of the key molecules and signalling pathways involved in the survival and maintenance of melanocyte stem cells (MSCs) in the hair follicle niche. Within the niche, hair follicle stem cells (HFSCs), MSCs themselves and the extracellular matrix are involved in the maintenance of MSCs’ homeostasis. *Notch*, *Wnt*, *TGFB*, *NFIB*, *EDNRB* and *CXCL12* signalling pathways are key pathways in the maintenance of homeostasis in MSCs.

Stem cells' crosstalk with adjacent cells and the niche microenvironment is vital for establishing, maintaining and activating stem cell fate, and follicular MSCs are no exception to this rule.^39^, ^40^, ^41^ MSCs lack multiple regulators that sustain the growth and development of the melanocytic lineage, but they still maintain homeostasis and play paramount roles in vivo. This is due to the ability of the niche in which MSCs reside to maintain the homeostasis of MSCs as well as promote the quiescence of MSCs to prevent their overgrowth.^23^ In this section, we discuss the key signalling pathways in the HF niche that maintain homeostasis in MSCs, including *Notch*, *Wnt*, *TGFB*, *NFIB*, *EDNRB* and *CXCL12* signalling pathways (Figure 3).

### 3.1 *Notch* signalling

The *Notch* signalling plays an indispensable role in the maintenance of MSCs in the HF.^42^ *Notch* interacts with its ligand to form the intracellular domain of Notch, which translocates to the nucleus to generate a transactivation complex with the transcription factor *RBPJ* to activate the transcription of downstream target genes like *HES1*.^43^, ^44^ Targeted *Notch*1 and *Notch*2 depletion results in the progressive loss of follicular MSCs with ageing and causes hair graying.^42^ Targeted deletion of the downstream transcription factor *RBPJ* results in similar hair graying by inducing apoptosis of melanoblasts and MSCs in the HF.^45^ *HES1* could suppress the initiation of apoptosis and preserve the survival of melanoblasts and MSCs by blocking the expression of genes required for melanoblast apoptosis.^45^ In addition, the absence of *RBPJ* also promotes premature maturation of MSCs to generate MCs in the lower part of the permanent portion of the HF.^46^ In short, the *Notch* signalling pathway maintains the homeostasis of melanoblasts and MSCs by inhibiting apoptosis and prevents MSCs from differentiating before reaching the hair bulb.

### 3.2 *Wnt/β‐catenin* signalling

*Wnt* signalling plays an essential role in the maintenance of follicular MSCs. *Wnt* suppresses the degradation of β‐catenin in MSCs cytoplasm by binding to *FZD* and *LRP5/6* receptors.^47^ Accumulated *β‐catenin* is translocated to the nucleus, where it interacts with the *T‐cell specific factor/lymphoid enhancer binding factor 1* transcription factor to bind to DNA to regulate the expression of downstream genes (e.g., *TYR*, *DCT*, etc.), promoting the proliferation and differentiation of MSCs into mature MCs.^47^, ^48^ Nevertheless, *Wnt/β‐catenin* signalling is not activated spontaneously by MSCs, and this process requires the assistance of other cells, such as HFSCs in the bulge or keratinocytes in the hair matrix.^49^, ^50^ Interestingly, *Wnt/β‐catenin* signalling is only active during the anagen, and it is turned off for the rest of the hair cycle.^48^ The inhibition of *Wnt* signalling is necessary to maintain MSCs in an undifferentiated state. High levels of *Wnt* inhibitors such as *DAB2*, *DKK3* and *SFRP1* are expressed in the HF niche, while MSCs themselves express *WIF1*, *DKK5*, *DAB2* and *SFRP1*.^49^, ^51^ These inhibitors inactivate *Wnt/β‐catenin* signalling and keep MSCs in an immature state. Downregulation of *Wnt* signalling promotes the de‐differentiation of MSCs, thus contributing to the maintenance of MSCs.^3^ In addition, downstream targets of the *Wnt* signalling, especially *MITF*, *PAX3* and *SOX10*, are implicated in the maintenance of MSCs in the niche.^52^ Collectively, these results indicate that inhibition of the *Wnt* signalling contributes to the maintenance of undifferentiated MSCs. Meanwhile, the importance of neighboring HFSCs in maintaining the immature state of MSCs was revealed.

### 3.3 *TGFB* signalling

*TGFB* signalling performed by HFSCs maintains the quiescence and homeostasis of MSCs by directly regulating the expression of cyclin and differentiation‐related genes.^53^ *TGFB* expression is decreased when MSCs are activated at the early anagen and increased when MSCs enter telogen.^53^ *TGFB* binds to its receptor and phosphorylates the downstream effector *SMAD2*, which not only induces the morphological transition of MCs to MSCs but also downregulates the expression of *MITF*, *PAX3*, and downstream melanogenic genes, so as to maintain the quiescence of MSCs and cause cell cycle arrest of MCs.^53^, ^54^ Deficiency of the *TGFBR2* leads to abnormal proliferation and differentiation of MSCs.^53^ These findings strongly imply that *TGFB* signalling plays a dual role in the maintenance of MSCs by inhibiting MSCs differentiation and inducing MSCs quiescence. However, the above‐described functions are dependent on the presence of *BCL2*. *BCL2*, a major regulator of apoptosis, serves an important anti‐apoptotic role in the maintenance and survival of MSCs.^53^ When *BCL2* is specifically deficient, *TGFB* signalling induces apoptosis in MSCs.^53^

HFSCs highly express *COL17A1*, a hemidesmosomal transmembrane protein attached to the epidermal basement membrane.^55^ *COL17A1* provides a framework structure for HF bulge to maintain the settlement of HFSCs and MSCs, and its defect leads to reduced anchoring of HFSCs and MSCs and atrophy of HF.^56^ Notably, *COL17A1* deficiency not only induces premature differentiation of MSCs but also promotes aberrant proliferation and differentiation of HFSCs, gradually losing their characteristics, such as impaired *TGFB* secretion, and then leading to the loss of MSCs.^53^ This suggests that *COL17A1* is required for MSCs’ maintenance by regulating *TGFB* expression.

### 3.4 *NFIB* signalling

*NFIB* is a transcription factor associated with cell development, cell regulation and stem cell maintenance.^57^ As the coordinator of behaviour between HFSCs and MSCs, *NFIB* has an important function in maintaining the homeostasis of MSCs.^58^ The conditional knockout of *NFIB* in HFSCs promotes the premature differentiation of MSCs and the uncoupling of MSCs and HFSCs, indicating that *NFIB* may be involved in the regulation of MSCs’ homeostasis.^58^ Intrinsically, *NFIB* serves as a gatekeeper to maintain the homeostasis of stem cells in the HF, and its loss enhances the self‐renewal of MSCs as well as disrupts epithelial–MSC synchronisation. Furthermore, *EDN2* is identified as a target of *NFIB*. By targeting the inhibition of *EDN2*, *NFIB* considerably prevents excessive proliferation and precocious differentiation of MSCs.^58^

### 3.5 *EDNRB* signalling

*EDNRB*, a G protein‐coupled receptor, is crucial for maintaining the proliferation and differentiation of MSCs.^59^ Conditional knockout of *EDNRB* resulted in a decrease in the absolute number of MSCs at the bulge and hair graying in mice, demonstrating the necessity of *EDNRB* for the maintenance of MSCs' homeostasis and proliferation.^60^ In addition, activation of *Wnt* signalling or the increase of *EDN1/2* could promote the proliferation and differentiation of MSCs by binding to *EDNRB*.^48^

### 3.6 *CXCL12* signalling

*CXCL12* is a chemokine secreted by HFSCs to modulate cell migration.^61^ *CXCL12* suppresses MSCs differentiation by binding to *CXCR7* on the surface of MSCs, dramatically lowering *MITF* mRNA expression that regulates MCs development and differentiation, and reducing nuclear translocation of *β‐catenin*.^62^ Besides, high *CXCL12* expression competitively inhibits stem cell factor (SCF), thus preventing MSCs from migrating out of the bulge.^62^ Taken as a whole, *CXCL12* attracts and targets MSCs in the proper position and facilitates the maintenance of MSCs in an undifferentiated state.

Recently, Wilson et al. reported that *BMI1* is an essential molecule for maintaining the stemness of MSCs.^63^ *BMI1* is a key molecule that regulates the self‐renewal of multiple adult stem cells, and its deletion leads to the progressive depletion of MSCs.^63^ These studies show that the crosstalk between follicular MSCs and their niche microenvironment plays an important regulatory role in the quiescent maintenance, proliferative differentiation and regenerative capacity of MSCs. HFSCs can precisely regulate the quiescent maintenance and proliferation differentiation of MSCs through specific molecular signals. In addition, since the follicular bulge is positioned in a complex microenvironment, its surrounding signals, such as keratinocytes, neurons, dermal tissues and adipose tissues, may modulate the homeostasis of MSCs.^64^, ^65^ For instance, the latest study revealed that SPRY1 protein loss in epidermal keratinocytes promotes MSCs migration to the epidermis in a p53/SCF/c‐Kit‐dependent manner, enriching the understanding of MSCs regulation by keratinocytes.^66^

## 4 ROLE OF MSCS IN SKIN DISEASES

### 4.1 Hair graying

**FIGURE 4:**
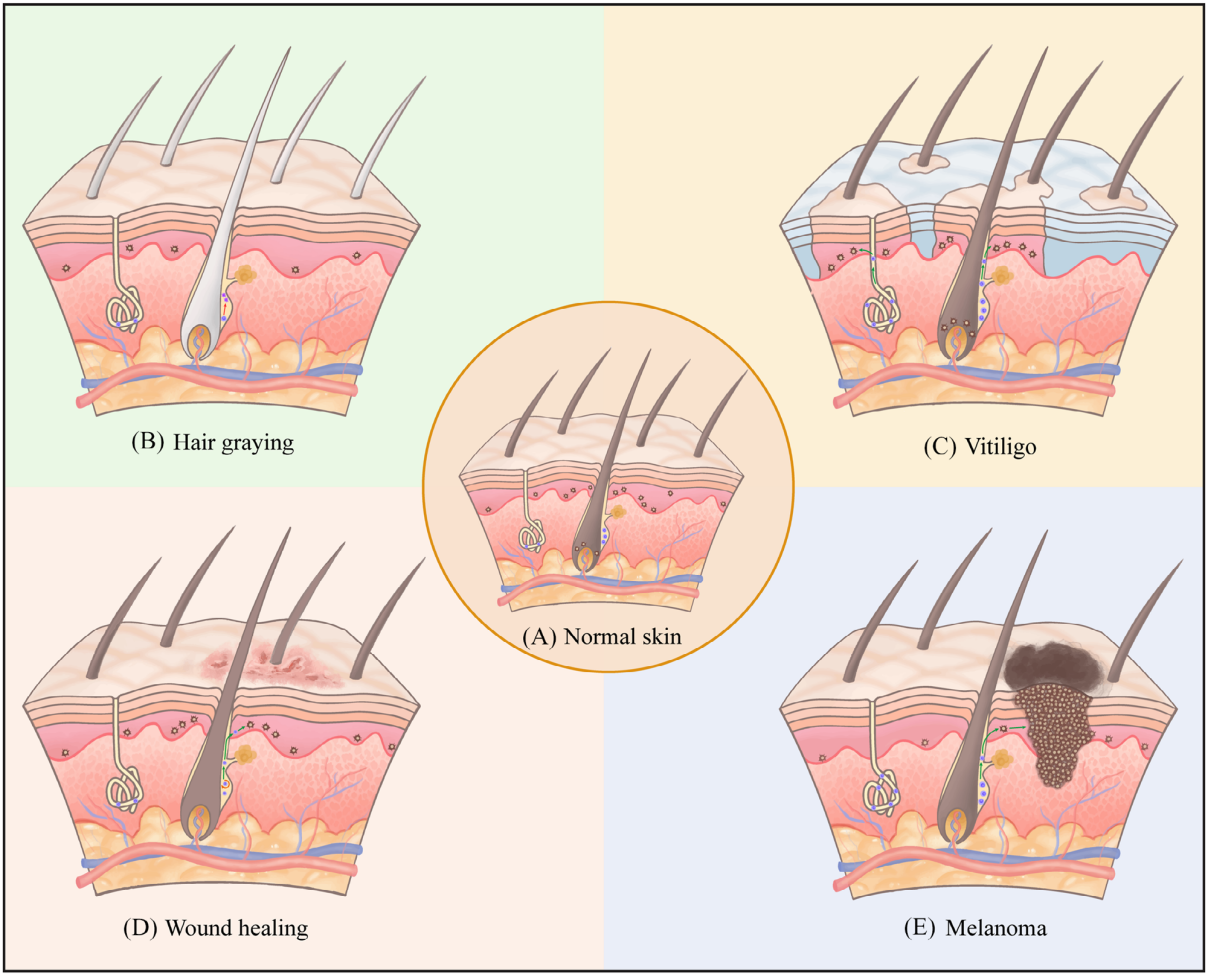
The role of melanocyte stem cells (MSCs) in different skin disorders: microstructural diagram. (A) Normal skin. MSCs (shown as blue dots) are located in the hair follicle bulge and exocrine sweat glands, and mature melanocytes (MCs) (shown as brown dots) are located in the hair matrix and epidermis. (B) Hair graying. The number of MSCs within the bulge is reduced or absent. The MSCs form ectopically differentiated melanocytes (shown as purple dots) that fail to provide functional melanocytes for the next hair cycle (shown as a red arrow), leading to the appearance of gray hair. (C) Vitiligo. Vitiligo lesions show a complete loss of MCs within the epidermis. However, intact MSCs are retained, and their number and distribution are similar to those of normal skin. Under the stimulation of certain specific factors, MSCs in hair follicles and exocrine sweat glands are activated to produce progeny MCs that migrate upward to the epidermis to complete the repigmentation of vitiligo (shown as a green arrow). (D) Skin wound healing. Under skin wounding such as UVB irradiation, follicular MSCs undergo self‐renewal (shown as an orange arrow), and then migrate to the epidermis to differentiate into functional MCs to provide a protective pigment barrier (shown as a green arrow). (E) Melanoma. Under the stimulation of risk factors, follicular MSCs are activated, migrate out of the bulge region, and differentiate into MCs in the epidermis (shown as a green arrow), further leading to melanogenesis (shown as a black cluster).

Hair graying is an obvious ageing‐associated phenotype. During the hair cycle, the reduction or absence of melanin synthesised by MCs in newborn hairs is considered to be responsible for gray hair formation.^67^ Therefore, as the source of MC replenishment, the role of MSCs in hair graying is of greater concern. Earlier, Nishimura et al. attributed the mechanism of age‐associated hair graying in mice and humans to incomplete maintenance of MSCs within the HF bulge.^68^ With physiological age, the number of MSCs in the bulge decreases or disappears, and ectopically differentiated MCs (EPM) emerge. MSCs lose their self‐renewal ability and fail to supply MCs for the next hair cycle, subsequently resulting in gray hair (Figure 4B).

In addition to physiological ageing, environmental insults and stress also induce MSCs depletion and hair graying. MSCs abrogation in mice can be observed in gray hair caused by ionising radiation or DNA‐damaging drugs.^69^, ^70^ For example, UVB irradiation induces *Wnt7a* secretion by mouse keratinocytes and promotes *β‐catenin* translocation into the nucleus. Activation of *Wnt/β‐catenin* signalling promotes MSCs differentiation, depletion and premature hair graying.^48^ However, unlike other stem cells that undergo apoptosis or senescence after DNA damage, this DNA damage response triggers MSCs to form EPM in the niche, which results in hair graying due to the defect in the self‐maintenance of MSCs (Figure 4B).^71^ The finding suggested that physiological gray hair may be related to the accumulation of DNA damage that occurs as people age.^71^, ^72^ In addition, Zhang et al. reported that acute stress promotes the release of norepinephrine from sympathetic nerve terminals innervating the HF bulge, which drives the rapid proliferation and differentiation of quiescent MSCs by binding to the *ADRB2* on the surface of MSCs, leading to permanent depletion of MSCs at the HF, which is considered to be the main reason of hair graying.^64^ This seems to suggest that MSCs depletion linked to long‐term accumulated stress may be the cause of physiological gray hair. Ex vivo cultured human follicular MSCs exposed to certain stress signalling mediators, such as ionising radiation, noradrenaline and hydrogen peroxide, exhibited ectopic pigmentation within the bulge similar to that observed in mouse models.^73^ This study supported the hypothesis that premature ectopic differentiation of MSCs is the key fate of human hair graying. Recently, Sun et al. pioneered the discovery that MSCs are retained in the follicular bulge during HF ageing, and their inability to de‐differentiate back to the HG is an important contributor to hair graying, suggesting that modulation of the motility of MSCs may be a new approach to preventing hair graying.^3^

Although there are many hypotheses about the mechanism of human hair graying, as the source of the HF pigment cycle, once the self‐renewal potential of MSCs is disrupted, the downstream melanocytic lineage will be irreversibly and completely lost. As a result, preventing MSCs depletion, suppressing MSCs ectopic differentiation and regulating the MSCs niche might be innovative avenues to prevent or reverse human hair graying.

### 4.2 Vitiligo

Vitiligo is a depigmented skin disorder caused by the destruction of epidermal MCs from a variety of aetiologies.^74^ Its typical clinical manifestation is progressive hypopigmentation of the skin. The pathological phenotype of vitiligo shows an eventual complete loss of MCs in the epidermis of vitiligo lesions.^75^ However, surprisingly, complete MSCs remain within the HF of the lesion areas and are similar to normal skin in number and distribution (Figure 4C).^76^ The appearance of black hairs in vitiligo lesions also confirms the existence of MSCs in the bulge, the reasoning behind which may be related to immune amnesty at the bulge site.^77^, ^78^ Song et al. recently showed that *ADRB2* expression was significantly elevated in the ORS of vitiligo patients, suggesting that norepinephrine binding to *ADRB2* may be involved in vitiligo‐associated gray hair formation by inhibiting the differentiation of follicular MSCs to mature MCs.^79^ Although MSCs may not be directly involved in vitiligo development, several studies have indicated that MSCs are the key and root cause of vitiligo repigmentation. Narrowband UVB (NB‐UVB) irradiation induces strong expression of epidermal *Wnt7a*, which promotes follicular MSCs to differentiate into mature MCs through activation of *β‐catenin* and migrate to the epidermis to complete repigmentation in mice.^33^ An elegant study showed that proper microinjury promotes the upward migration of quiescent MSCs in the mouse HF to repopulate epidermal MCs by activating the *Wnt/β‐catenin* signalling, promoting vitiligo repigmentation (Figure 4C).^80^ It was previously considered that vitiligo in hairless areas such as the palmoplantar and mucous membranes was difficult to recover. The ‘medium‐sized spot repigmentation pattern’ revealed, however, that MSCs also exist in small sweat glands and renew themselves under stress, producing progeny MCs for epidermal repigmentation (Figure 4C).^38^ Furthermore, intervening in MSC‐associated signalling pathways may promote vitiligo repigmentation. For example, *Wnt/β‐catenin* signalling not only prevents the progression of active vitiligo but also promotes white patch pigmentation.^81^ These results suggest that drugs or compounds with the function of activating *Wnt/β‐catenin* signalling may be potential candidates for the development of new therapeutic agents for vitiligo.

Furthermore, other skin cells neighbouring MSCs, including keratinocytes, fibroblasts and endothelial cells, may play an important role in pigmentation by regulating MSCs.^82^ For example, UV irradiation may promote the secretion of cytokines and paracrine growth factors (e.g., α‐melanocyte‐stimulating hormone [α‐MSH], endothelin‐1, etc.) by keratinocytes through the activation of *p53*, which may in turn regulate the migration, proliferation, and differentiation of MSCs, thus contributing to the repigmentation of vitiligo.^82^, ^83^ The binding of α‐MSH to the melanocortin‐1 receptor (MC1R) is an important signal for melanogenesis. Afamelanotide, an α‐MSH analogue, synergistically with NB‐UVB could induce the migration and differentiation of follicular MSCs into epidermal MCs, which has shown promise in the clinical treatment of vitiligo (NCT04525157 and NCT01430195).^84^, ^85^, ^86^ Phase II clinical studies evaluating the pigmentation and safety of subcutaneous bioresorbable Afamelanotide implants for the treatment of facial vitiligo are currently underway (NCT05210582). Furthermore, clinical trials of non‐cultured ORS HF cell suspension transplantation containing MSCs have shown favorable skin pigmentation in patients with stable vitiligo (NCT01923142).^87^, ^88^ These findings show great promise for the application of MSCs in vitiligo clinical treatment.

Collectively, the number, self‐renewal ability and niche microenvironment of MSCs are the pivotal factors affecting vitiligo repigmentation. Intervention of aberrant stem cell‐associated regulatory factors or signals, reconstruction of the niche microenvironment, or remodelling of the crosstalk of MSCs and HFSCs would be a milestone advancement for the biological therapy of vitiligo patients.

### 4.3 Skin wound healing

Wound healing is a comprehensive and highly coordinated procedure.^89^ Epidermal wound healing is mainly dependent on the proliferation and differentiation of various skin stem cells, which supply functional cells for tissue regeneration.^90^ In particular, stem cells at the bulge are considered to represent the ultimate source of the skin epithelial cell lineage for their potential to reconstruct the HF and epidermis.^12^ Chou et al. demonstrated that under UVB‐induced skin wounding, mouse follicular MSCs migrated directly to the epidermis via the MC1R–ACTH–α‐MSH pathway to differentiate into mature epidermal MCs to provide a pigmented protective barrier and promote wound repair.^91^ Although this direct migration pattern led to a decrease in the number of MSCs in the niche, interestingly, the appearance of gray hair was not observed.^91^ This indicates that the self‐maintenance of MSCs is prioritised over the differentiation of MSCs (Figure 4D).

However, in contrast to follicular MSCs at wound sites, which preferentially differentiate early in wound healing to maintain skin integrity, at non‐wound sites, MSCs appear to function only during the later phases of wound healing.^92^ This is probably a protective mechanism. In the early phases of wound healing, quiescence of the bulge region at uninjured sites is important to preserve the regenerative potential of the HF, which might otherwise be depleted by the regenerative potential of stem cells within the bulge after wound healing. Simultaneously, this greatly prevents the tumorigenesis induced by the injury. Activation of *β‐catenin* induces MSCs to proliferate and differentiate into epidermal MCs to contribute to epidermal pigmentation during the skin wound healing phase in the mouse.^48^ These findings demonstrate the incredibly important role played by MSCs in skin wound healing and provide new perspectives for the clinical management of post‐traumatic pigment disorders.

### 4.4 Melanoma

Melanoma is a highly aggressive malignancy in the lineage of MCs, and its origin and pathogenesis have not been fully elucidated. Due to their longevity, MSCs accumulate enormous levels of DNA damage during senescence, making them the most likely source of melanoma stem cells.^93^ In the *Tyr‐CreER:Braf:Pten* mouse model, Moon et al. demonstrated that UVB induces the activation and translocation of MSCs in the HF in an inflammatory and dose‐dependent manner, promoting melanogenesis.^94^ Nevertheless, since *Tyr‐CreER* targets both MSCs in the HF and MCs in the epidermis, it is intrinsically difficult to determine the true origin of melanoma using *Tyr‐CreER*.^95^, ^96^ Subsequently, Sun et al. constructed a *c‐Kit‐CreER* mouse model targeting MSCs only, clearly showing that oncogenic MCs derived from MSCs invade the underlying dermis and form heterogeneous melanoma that closely resembles human melanoma.^97^ In contrast to Moon et al., who claimed that UVB is required to elicit melanoma, Sun et al. proved that MSCs can initiate epidermal melanoma during normal hair regeneration.^94^, ^97^ Remarkably, Eshiba et al. successfully established a *DCT‐CreER KI; Braf‐CA;Pten‐fx/fx* mouse model with a fate‐tracing system for MSCs, demonstrating not only that MSCs from exocrine sweat glands are the origin of melanogenesis but also mimicking the formation of human acral melanoma.^18^ Stimulated by UV irradiation or *Wnt* or *EDN* secreted by the epidermal microenvironment increased the susceptibility of MSCs to BRAF‐V600E transformation, which promoted melanomagenesis in the mouse.^98^, ^99^

While these findings confirmed that MSCs are the cellular origin of melanoma, surprisingly, human melanoma is uncommon in HFs with rich MSCs. Indeed, melanoma does not grow in quiescent MSCs in the HF bulge, but it does develop once MSCs are activated and move out of the bulge (Figure 4E).^94^, ^100^ Similarly, in response to stimulation by melanoma risk factors, quiescent MSCs in exocrine sweat glands undergo self‐renewal and provide progeny cells to the epidermis through the ducts, resulting in epidermal melanoma.^18^ This explains the characteristic of a dermoscopy in a ‘parallel ridge pattern’ of acral melanoma.^38^ Crucially, these phenomena suggest that the niche in which MSCs reside maintains the quiescence of MSCs and has an inhibitory effect on melanogenesis.^98^

In recent years, melanoma patients have developed acquired resistance to targeted therapies and immunotherapy, and new therapeutic options are urgently needed. Any factor that affects the development of MSCs and their subsequent processes has the potential to modulate melanogenesis. As a result, a better understanding of the development of MSCs, the regulation of melanogenesis inhibition by the niche in which MSCs reside, and the exogenous stimulation pathways that activate MSCs might open new possibilities for humanity to reconsider the origin and nature of melanoma and design efficient clinical treatment strategies.

## 5 TECHNOLOGICAL ADVANCES IN MSCS RESEARCH

Lineage tracking is one of the key components of MSCs research. Compared with tissue sections that only show the status of MSCs at a specific moment in time, 3D in vivo imaging can track the migration and differentiation of MSCs in real time, providing an excellent tool for defining the migration pathways of MSCs in vivo.^3^ Moreover, two‐photon microscopy with high depth penetration, low invasiveness and precise spatial point focusing has recently been used for MSCs lineage tracking.^101^

Single‐cell RNA sequencing (scRNA‐seq) has created new opportunities for research in various fields due to its crucial role in the discovery of new cell populations, dynamic observation of developmental processes and revelation of gene regulatory mechanisms in recent years.^102^, ^103^ Utilising 3D imaging and scRNA‐seq to track the process of cellular senescence and movement within each HF in real time, Sun et al. found that MSCs are trapped in the bulge resulting in a loss of stem cell stemness, providing a new perspective on reverse hair graying.^3^ This finding provided a fresh perspective on reverse hair graying. The scRNA‐seq technology may help us to discover new cellular markers and subpopulations, and identify key signalling pathways and transcription factors involved in MSCs self‐renewal and differentiation.

CRISPR/Cas9 is a novel and efficient targeted gene editing tool. The combination of CRISPR/Cas9 technology with MSCs could provide an excellent model for dynamic observation of somatic cell development, characterisation and fate tracing.^104^, ^105^ Meanwhile, using CRISPR/Cas9 technology, specific disease models involving MSCs can be constructed to explore the molecular mechanisms of diseases and develop potential therapeutic strategies. In the future, it is anticipated that CRISPR/Cas9 technology will greatly improve the treatment of disorders associated with MSCs due to its advantages in precision, stability and simplicity.^106^

The development of practical models of MSCs has provided researchers with tools to study MSCs in vitro and in vivo (Table 2). These models include genetically engineered mouse models,^18^, ^30^, ^31^, ^33^, ^48^, ^66^, ^91^, ^94^, ^96^, ^97^, ^101^ zebrafish models,^107^ human vitiligo skin biopsies^22^ and human pluripotent stem cell models.^24^ These models serve as important tools for studying the biological properties, differentiation potential, disease mechanisms and potential therapeutic strategies of MSCs. Therefore, the development and optimisation of MSCs models are of great scientific and clinical value for the development of skin biology and pigmentation diseases.

**TABLE 2:** Practical models that were used for melanocyte stem cells (MSCs) study.

Model	Characteristic	Contribution	Ref.
K14‐SLF; DCT‐lacZ mice	Expressed SLF in the epidermis Labelling melanoblasts, MSCs and MCs	Follicular MSCs represent the reservoir of epidermal MCs, and the expression of SLF promotes follicular MSCs migration to the epidermis.	^30^
Tyr‐CreER mice	Labelling melanoblasts, MSCs and MCs	A useful resource for assessing the characterisation of MSCs function and dynamics and for constructing mouse models of malignant melanoma is provided.	^96^
HR‐1×HR/De F1 mice	The presence of epidermal MCs in dorsal skin A suitable animal model to study the molecular mechanism underlying UV‐induced epidermal pigmentation	UVB irradiation induces the proliferation and differentiation of bulge MSCs into melanoblasts, which migrate to the epidermis to differentiate into MCs.	^33^
MC1R^e/e^ mice	Expressing non‐functional MC1R	Follicular MSCs differentiate into functional epidermal MCs in an MC1R‐dependent manner.	^91^
DCT‐H2B‐GFP mice	Labelling melanoblasts, MSCs and MCs	Sweat glands are identified as an anatomical ecological niche for MSCs in mammalian palmar skin.	^38^
Tyr‐CreER^T2^; β‐catenin^fl(ex3)/+^ mice	Labelling melanoblasts, MSCs and MCs Expressed β‐catenin dominant mutant in MCs	Wnt signalling induces MSCs to differentiate into pigment‐generating MCs.	^48^
TBP mice	Labelling melanoblasts, MSCs and MCs Expressed BrafV600E and loss of Pten function	MSCs are the origin cells of melanoma, and MSCs quiescence is the inhibitor of melanoma genesis.	^94^
c‐Kit‐CreER mice	Labelling MSCs	A mouse model targeting only MSCs is established.	^97^
c‐Kit‐CreER:Braf:Pten mice	Labelling MSCs Expressed BrafV600E Deletion of Pten in c‐Kit^+^ MSCs	The model demonstrates that MSCs are the true origin of melanoma and mimics the progression of human melanoma from the epidermis to the dermis.	^97^
DBP mice	Labelling melanoblasts, MSCs and MCs Expressed BrafV600E and loss of Pten function	The model can be used to construct a mouse acral melanoma model and mimic the cellular dynamics of MSCs proliferation in human acral melanoma.	^18^
B6‐DCT‐H2BGFP mice	Expression of GFP in all melanocytic lineage cells Regulation of GFP expression by doxycycline	The model can be used for in vivo studies of MCs requiring a defined genetic background.	^31^
K14‐SPRY1 mice	Epidermal keratinocyte‐specific lack of SPRY1	Epidermal keratinocyte SPRY1 loss induces follicular MSCs to migrate to the epidermis in a p53/SCF/c‐KIT‐dependent manner.	^66^, ^101^
DCT‐rtTA; Tre‐H2B‐GFP mice	Labelling MSCs	Using this model, important cellular and molecular players that promote MSCs proliferation and epidermal repopulation under UVB irradiation were identified, and it was determined that cyclooxygenase signalling or prostaglandin E2 supplementation significantly enhanced this process.	
MITFa‐hBRAF^V600E^Tomato p53^−/−^ zebrafish	MITFa as melanoblast promoter Zebrafish expressing BRAF^V600E^ and p53 mutations	Melanoblasts are one of the origins of melanoma.	^107^
Human vitiligo skin biopsies	Analysis of MSCs, melanoblasts and MCs in human hair follicles and epidermis	The human model supports the concept that the follicle bulge is the location of MSCs that are the major precursors for epidermal MCs repopulation.	^22^
hiMels	High sequence similarity to human epidermal MCs	The long‐term functions of hiMels in vivo are to reconstitute pigmented hair follicles and integrate into normal regions for both mature MCs and MSCs.	^24^

Abbreviations: DBP, DCT‐CreER KI; Braf‐CA;Pten‐fx/fx; DCT, dopachrome tautomerase; GFP, green fluorescent protein; hiMels, human induced pluripotent stem cell‐derived melanocytes; MCs, melanocytes; MC1R, melanocortin 1 receptor; SLF, stem cell factor; TBP, Tyr‐CreER; LSL‐BRAF^V600E^; Ptenflox/flox; Tyr, tyrosinase.

Additionally, recently, HF structures constructed entirely from human primary cells have been successfully fabricated using 3D bioprinting technology.^108^ Three‐dimensional bioprinting technology can create models with complex structures, which lay the foundation for further research on the differentiation process and biological characteristics of MSCs. Meanwhile, 3D bioprinting technology can also be employed to construct disease models such as vitiligo, which can help to study the role of MSCs in the pathogenesis and treatment of the disease.

In summary, 3D in vivo imaging, two‐photon microscopy, scRNA‐seq, CRISPR/Csa9, 3D bioprinting technology and practical models provide powerful tools for MSCs research, which can help deepen our understanding of MSCs and advance the development of regenerative medicine and therapeutic strategies for skin pigmentation disorders.

## 6 CONCLUSION AND PERSPECTIVES

MSCs located in HFs, epidermis or eccrine sweat glands play a determinant role in maintaining hair and skin pigmentation and important skin functions. The intricate interaction between MSCs and niche provides significant views into skin biology development. More importantly, this review provides fresh insights into the prevention and treatment of gray hair, depigmentation disorders, skin wound healing and melanoma through an intensive exploration of MSCs.

Since MSCs are long‐lived, multipotent and highly manipulable in vivo and in vitro, they may provide a source of stem cells for the treatment of diverse skin pigment disorders.^109^ However, research in this field is still in its infancy due to technical limitations and challenges with sample collection. Technologies such as genomics studies, scRNA‐seq and CRISPR/Csa9 may provide new approaches to tackle these challenges.^110^ By using human‐derived MSCs and gene editing technology, specific disease models can be constructed, which will help better explore the unknown biological features of human MSCs and provide a theoretical foundation for clinically personalised and precise treatment. Exploiting more human‐based methodologies will help us to elucidate the uncharted territories of MSCs that have yet to be identified. In addition, the safety, efficacy and side effects of MSCs in clinical treatment need to be further evaluated.

However, despite the great progress that has been made in the study of MSCs, there are still many problems that have not been fully explored. For example, MSCs may be only one important part of the regeneration and differentiation of MCs, while the role of other precursor cells in the melanocytic lineage on the development and differentiation of MCs needs to be further investigated. In depigmentation diseases, how to effectively activate MSCs and make them play an appropriate and lasting role is a tricky problem that needs to be explored in the future. Similar hair graying is observed, but the difference in the repigmentation rate of gray hair between alopecia areata and vitiligo may suggest different pathogenesis and characteristics of MSCs. In addition, whether extrafollicular MSCs can be recruited to the bulge to promote gray hair repigmentation requires further investigation.

As a result, it is of great significance to further elucidate the specific signals and mechanisms of crosstalk among human MSCs and apply scRNA‐seq, gene editing technology, spatial transcriptomics and whole‐genome sequencing in MSCs field, which will help establish a new paradigm for skin regeneration research. It is believed that in the near future, the repigmentation of skin and hair will become the pioneers of stem cell transformation, providing the theoretical and practical cornerstones for regenerative medicine in the regeneration and repair of various tissues and organs.

## AUTHOR CONTRIBUTIONS

Luling Huang, Shuli Li and Chunying Li contributed to the conception, design and final approval of the submitted version. Luling Huang and Yuzhi Zuo contributed to manuscript writing. All authors have read and approved the final manuscript.

## CONFLICT OF INTEREST STATEMENT

The authors declare they have no conflicts of interest.

## ETHICS STATEMENT

Not applicable.

## Data Availability

Data sharing is not applicable to this article since no new data were created or analysed in this study.

## References

[1] García‐Castro M , Bronner‐Fraser M . Induction and differentiation of the neural crest. Curr Opin Cell Biol. 1999;11(6):695‐698. 10600707 10.1016/s0955-0674(99)00038-1

[2] Serbedzija GN , Fraser SE , Bronner‐Fraser M . Pathways of trunk neural crest cell migration in the mouse embryo as revealed by vital dye labelling. Development. 1990;108(4):605‐612. 2387238 10.1242/dev.108.4.605

[3] Sun Q , Lee W , Hu H , et al. Dedifferentiation maintains melanocyte stem cells in a dynamic niche. Nature. 2023;616(7958):774‐782. 37076619 10.1038/s41586-023-05960-6 PMC10132989

[4] Li L , Clevers H . Coexistence of quiescent and active adult stem cells in mammals. Science. 2010;327(5965):542‐545. 20110496 10.1126/science.1180794 PMC4105182

[5] Cotsarelis G , Sun TT , Lavker RM . Label‐retaining cells reside in the bulge area of pilosebaceous unit: implications for follicular stem cells, hair cycle, and skin carcinogenesis. Cell. 1990;61(7):1329‐1337. 2364430 10.1016/0092-8674(90)90696-c

[6] Schmidt‐Ullrich R , Paus R . Molecular principles of hair follicle induction and morphogenesis. Bioessays. 2005;27(3):247‐261. 15714560 10.1002/bies.20184

[7] Li A . The biology of melanocyte and melanocyte stem cell. Acta Biochim Biophys Sin (Shanghai). 2014;46(4):255‐260. 24449785 10.1093/abbs/gmt145

[8] Adameyko I , Lallemend F , Aquino JB , et al. Schwann cell precursors from nerve innervation are a cellular origin of melanocytes in skin. Cell. 2009;139(2):366‐379. 19837037 10.1016/j.cell.2009.07.049

[9] Nitzan E , Pfaltzgraff ER , Labosky PA , Kalcheim C . Neural crest and Schwann cell progenitor‐derived melanocytes are two spatially segregated populations similarly regulated by Foxd3. Proc Natl Acad Sci U S A. 2013;110(31):12709‐12714. 23858437 10.1073/pnas.1306287110 PMC3732929

[10] Blanpain C , Fuchs E . Epidermal homeostasis: a balancing act of stem cells in the skin. Nat Rev Mol Cell Biol. 2009;10(3):207‐217. 19209183 10.1038/nrm2636 PMC2760218

[11] Blanpain C , Sotiropoulou PA . A dominant role of the hair follicle stem cell niche in regulating melanocyte stemness. Cell Stem Cell. 2010;6(2):95‐96. 20144781 10.1016/j.stem.2010.01.006

[12] Myung P , Ito M . Dissecting the bulge in hair regeneration. J Clin Invest. 2012;122(2):448‐454. 22293183 10.1172/JCI57414 PMC3266778

[13] Horikawa T , Norris DA , Johnson TW , et al. DOPA‐negative melanocytes in the outer root sheath of human hair follicles express premelanosomal antigens but not a melanosomal antigen or the melanosome‐associated glycoproteins tyrosinase, TRP‐1, and TRP‐2. J Invest Dermatol. 1996;106(1):28‐35. 8592077 10.1111/1523-1747.ep12326989

[14] Narisawa Y , Kohda H , Tanaka T . Three‐dimensional demonstration of melanocyte distribution of human hair follicles: special reference to the bulge area. Acta Derm Venereol. 1997;77(2):97‐101. 9111816 10.2340/000155557797101

[15] Lin JY , Fisher DE . Melanocyte biology and skin pigmentation. Nature. 2007;445(7130):843‐850. 17314970 10.1038/nature05660

[16] Li A , Ma Y , Jin M , et al. Activated mutant NRas(Q61K) drives aberrant melanocyte signaling, survival, and invasiveness via a Rac1‐dependent mechanism. J Invest Dermatol. 2012;132(11):2610‐2621. 22718121 10.1038/jid.2012.186 PMC3472562

[17] Nakamura M , Fukunaga‐Kalabis M , Yamaguchi Y , et al. Site‐specific migration of human fetal melanocytes in volar skin. J Dermatol Sci. 2015;78(2):143‐148. 25818865 10.1016/j.jdermsci.2015.03.003

[18] Eshiba S , Namiki T , Mohri Y , et al. Stem cell spreading dynamics intrinsically differentiate acral melanomas from nevi. Cell Rep. 2021;36(5):109492. 34348144 10.1016/j.celrep.2021.109492

[19] Ikeda Y , Wada A , Hasegawa T , Yokota M , Koike M , Ikeda S . Melanocyte progenitor cells reside in human subcutaneous adipose tissue. PLoS One. 2021;16(8):e0256622. 34432824 10.1371/journal.pone.0256622 PMC8386863

[20] Ueno M , Aoto T , Mohri Y , Yokozeki H , Nishimura EK . Coupling of the radiosensitivity of melanocyte stem cells to their dormancy during the hair cycle. Pigment Cell Melanoma Res. 2014;27(4):540‐551. 24730534 10.1111/pcmr.12251

[21] Harris ML , Buac K , Shakhova O , et al. A dual role for SOX10 in the maintenance of the postnatal melanocyte lineage and the differentiation of melanocyte stem cell progenitors. PLoS Genet. 2013;9(7):e1003644. 23935512 10.1371/journal.pgen.1003644 PMC3723529

[22] Goldstein NB , Koster MI , Hoaglin LG , et al. Narrow band ultraviolet B treatment for human vitiligo is associated with proliferation, migration, and differentiation of melanocyte precursors. J Invest Dermatol. 2015;135(8):2068‐2076. 25822579 10.1038/jid.2015.126 PMC4683025

[23] Osawa M , Egawa G , Mak SS , et al. Molecular characterization of melanocyte stem cells in their niche. Development. 2005;132(24):5589‐5599. 16314490 10.1242/dev.02161

[24] Liu LP , Li YM , Guo NN , et al. Therapeutic potential of patient iPSC‐derived iMelanocytes in autologous transplantation. Cell Rep. 2019;27(2):455‐466.e455. 30970249 10.1016/j.celrep.2019.03.046

[25] Nishimura EK . Melanocyte stem cells: a melanocyte reservoir in hair follicles for hair and skin pigmentation. Pigment Cell Melanoma Res. 2011;24(3):401‐410. 21466661 10.1111/j.1755-148X.2011.00855.x

[26] Nishikawa‐Torikai S , Osawa M , Nishikawa S . Functional characterization of melanocyte stem cells in hair follicles. J Invest Dermatol. 2011;131(12):2358‐2367. 21753783 10.1038/jid.2011.195

[27] Yamada T , Hasegawa S , Inoue Y , et al. Comprehensive analysis of melanogenesis and proliferation potential of melanocyte lineage in solar lentigines. J Dermatol Sci. 2014;73(3):251‐257. 24314758 10.1016/j.jdermsci.2013.11.005

[28] Joshi SS , Tandukar B , Pan L , et al. CD34 defines melanocyte stem cell subpopulations with distinct regenerative properties. PLoS Genet. 2019;15(4):e1008034. 31017901 10.1371/journal.pgen.1008034 PMC6481766

[29] Brombin A , Simpson DJ , Travnickova J , et al. Tfap2b specifies an embryonic melanocyte stem cell that retains adult multifate potential. Cell Rep. 2022;38(2):110234. 35021087 10.1016/j.celrep.2021.110234 PMC8764619

[30] Nishimura EK , Jordan SA , Oshima H , et al. Dominant role of the niche in melanocyte stem‐cell fate determination. Nature. 2002;416(6883):854‐860. 11976685 10.1038/416854a

[31] Tandukar B , Kalapurakal E , Hornyak TJ . B6‐Dct‐H2BGFP bitransgenic mice: a standardized mouse model for in vivo characterization of melanocyte development and stem cell differentiation. Pigment Cell Melanoma Res. 2021;34(5):905‐917. 33544968 10.1111/pcmr.12959

[32] Mak SS , Moriyama M , Nishioka E , Osawa M , Nishikawa S . Indispensable role of Bcl2 in the development of the melanocyte stem cell. Dev Biol. 2006;291(1):144‐153. 16427619 10.1016/j.ydbio.2005.12.025

[33] Yamada T , Hasegawa S , Inoue Y , et al. Wnt/beta‐catenin and kit signaling sequentially regulate melanocyte stem cell differentiation in UVB‐induced epidermal pigmentation. J Invest Dermatol. 2013;133(12):2753‐2762. 23702581 10.1038/jid.2013.235

[34] Wang Y , Li S , Li C . Clinical features, immunopathogenesis, and therapeutic strategies in vitiligo. Clin Rev Allergy Immunol. 2021;61(3):299‐323. 34283349 10.1007/s12016-021-08868-z

[35] Singh C , Parsad D , Kanwar AJ , Dogra S , Kumar R . Comparison between autologous noncultured extracted hair follicle outer root sheath cell suspension and autologous noncultured epidermal cell suspension in the treatment of stable vitiligo: a randomized study. Br J Dermatol. 2013;169(2):287‐293. 23517382 10.1111/bjd.12325

[36] Parsad D , Pandhi R , Dogra S , Kumar B . Clinical study of repigmentation patterns with different treatment modalities and their correlation with speed and stability of repigmentation in 352 vitiliginous patches. J Am Acad Dermatol. 2004;50(1):63‐67. 14699367 10.1016/s0190-9622(03)00786-2

[37] Davids LM , du Toit E , Kidson SH , Todd G . A rare repigmentation pattern in a vitiligo patient: a clue to an epidermal stem‐cell reservoir of melanocytes? Clin Exp Dermatol. 2009;34(2):246‐248. 18828846 10.1111/j.1365-2230.2008.02793.x

[38] Okamoto N , Aoto T , Uhara H , et al. A melanocyte–melanoma precursor niche in sweat glands of volar skin. Pigment Cell Melanoma Res. 2014;27(6):1039‐1050. 25065272 10.1111/pcmr.12297

[39] Clevers H , Loh KM , Nusse R . Stem cell signaling. An integral program for tissue renewal and regeneration: wnt signaling and stem cell control. Science. 2014;346(6205):1248012. 25278615 10.1126/science.1248012

[40] Moore KA , Lemischka IR . Stem cells and their niches. Science. 2006;311(5769):1880‐1885. 16574858 10.1126/science.1110542

[41] Lei M , Chuong CM . Stem cells. Aging, alopecia, and stem cells. Science. 2016;351(6273):559‐560. 26912687 10.1126/science.aaf1635

[42] Kumano K , Masuda S , Sata M , et al. Both Notch1 and Notch2 contribute to the regulation of melanocyte homeostasis. Pigment Cell Melanoma Res. 2008;21(1):70‐78. 18353145 10.1111/j.1755-148X.2007.00423.x

[43] Schouwey K , Aydin IT , Radtke F , Beermann F . RBP‐Jκ‐dependent Notch signaling enhances retinal pigment epithelial cell proliferation in transgenic mice. Oncogene. 2011;30(3):313‐322. 20856205 10.1038/onc.2010.428

[44] Borggrefe T , Oswald F . The Notch signaling pathway: transcriptional regulation at Notch target genes. Cell Mol Life Sci. 2009;66(10):1631‐1646. 19165418 10.1007/s00018-009-8668-7 PMC11115614

[45] Moriyama M , Osawa M , Mak SS , et al. Notch signaling via Hes1 transcription factor maintains survival of melanoblasts and melanocyte stem cells. J Cell Biol. 2006;173(3):333‐339. 16651378 10.1083/jcb.200509084 PMC2063834

[46] Aubin‐Houzelstein G , Djian‐Zaouche J , Bernex F , et al. Melanoblasts' proper location and timed differentiation depend on Notch/RBP‐J signaling in postnatal hair follicles. J Invest Dermatol. 2008;128(11):2686‐2695. 18463680 10.1038/jid.2008.120

[47] Nusse R , Clevers H . Wnt/β‐catenin signaling, disease, and emerging therapeutic modalities. Cell. 2017;169(6):985‐999. 28575679 10.1016/j.cell.2017.05.016

[48] Rabbani P , Takeo M , Chou W , et al. Coordinated activation of Wnt in epithelial and melanocyte stem cells initiates pigmented hair regeneration. Cell. 2011;145(6):941‐955. 21663796 10.1016/j.cell.2011.05.004 PMC3962257

[49] Lim X , Tan SH , Yu KL , Lim SB , Nusse R . Axin2 marks quiescent hair follicle bulge stem cells that are maintained by autocrine Wnt/β‐catenin signaling. Proc Natl Acad Sci U S A. 2016;113(11):E1498‐E1505. 26903625 10.1073/pnas.1601599113 PMC4801317

[50] Masunaga T , Shimizu H , Yee C , et al. The extracellular domain of BPAG2 localizes to anchoring filaments and its carboxyl terminus extends to the lamina densa of normal human epidermal basement membrane. J Invest Dermatol. 1997;109(2):200‐206. 9242508 10.1111/1523-1747.ep12319337

[51] Nishikawa S , Osawa M . Generating quiescent stem cells. Pigment Cell Res. 2007;20(4):263‐270. 17630959 10.1111/j.1600-0749.2007.00388.x

[52] Kubic JD , Young KP , Plummer RS , Ludvik AE , Lang D . Pigmentation PAX‐ways: the role of Pax3 in melanogenesis, melanocyte stem cell maintenance, and disease. Pigment Cell Melanoma Res. 2008;21(6):627‐645. 18983540 10.1111/j.1755-148X.2008.00514.x PMC2979299

[53] Nishimura EK , Suzuki M , Igras V , et al. Key roles for transforming growth factor beta in melanocyte stem cell maintenance. Cell Stem Cell. 2010;6(2):130‐140. 20144786 10.1016/j.stem.2009.12.010 PMC3437996

[54] Massagué J . TGFbeta in cancer. Cell. 2008;134(2):215‐230. 18662538 10.1016/j.cell.2008.07.001 PMC3512574

[55] Franzke CW , Tasanen K , Schumann H , Bruckner‐Tuderman L . Collagenous transmembrane proteins: collagen XVII as a prototype. Matrix Biol. 2003;22(4):299‐309. 12935815 10.1016/s0945-053x(03)00051-9

[56] Matsumura H , Mohri Y , Binh NT , et al. Hair follicle aging is driven by transepidermal elimination of stem cells via COL17A1 proteolysis. Science. 2016;351(6273):aad4395. 26912707 10.1126/science.aad4395

[57] Adam RC , Yang H , Ge Y , et al. NFI transcription factors provide chromatin access to maintain stem cell identity while preventing unintended lineage fate choices. Nat Cell Biol. 2020;22(6):640‐650. 32393888 10.1038/s41556-020-0513-0 PMC7367149

[58] Chang CY , Pasolli HA , Giannopoulou EG , et al. NFIB is a governor of epithelial‐melanocyte stem cell behaviour in a shared niche. Nature. 2013;495(7439):98‐102. 23389444 10.1038/nature11847 PMC3635831

[59] Li H , Fan L , Zhu S , et al. Epilation induces hair and skin pigmentation through an EDN3/EDNRB‐dependent regenerative response of melanocyte stem cells. Sci Rep. 2017;7(1):7272. 28779103 10.1038/s41598-017-07683-x PMC5544680

[60] Takeo M , Lee W , Rabbani P , et al. EdnrB governs regenerative response of melanocyte stem cells by crosstalk with Wnt signaling. Cell Rep. 2016;15(6):1291‐1302. 27134165 10.1016/j.celrep.2016.04.006 PMC5391032

[61] Belmadani A , Jung H , Ren D , Miller RJ . The chemokine SDF‐1/CXCL12 regulates the migration of melanocyte progenitors in mouse hair follicles. Differentiation. 2009;77(4):395‐411. 19281787 10.1016/j.diff.2008.10.015 PMC4461245

[62] Yamada T , Hasegawa S , Hasebe Y , et al. CXCL12 regulates differentiation of human immature melanocyte precursors as well as their migration. Arch Dermatol Res. 2019;311(1):55‐62. 30483878 10.1007/s00403-018-1880-2

[63] Wilson MM , Danielian PS , Salus G , Ferretti R , Whittaker CA , Lees JA . BMI1 is required for melanocyte stem cell maintenance and hair pigmentation. Pigment Cell Melanoma Res. 2023;36(5):399‐406. 37132544 10.1111/pcmr.13088 PMC11344272

[64] Zhang B , Ma S , Rachmin I , et al. Hyperactivation of sympathetic nerves drives depletion of melanocyte stem cells. Nature. 2020;577(7792):676‐681. 31969699 10.1038/s41586-020-1935-3 PMC7184936

[65] Chueh SC , Lin SJ , Chen CC , et al. Therapeutic strategy for hair regeneration: hair cycle activation, niche environment modulation, wound‐induced follicle neogenesis, and stem cell engineering. Expert Opin Biol Ther. 2013;13(3):377‐391. 23289545 10.1517/14712598.2013.739601 PMC3706200

[66] Cui YZ , Xu F , Zhou Y , et al. SPRY1 deficiency in keratinocytes induces follicular melanocyte stem cells migration to epidermis through p53/SCF/C‐KIT signaling. J Invest Dermatol. 2024. 10.1016/j.jid.2024.02.018 38462125

[67] O'Sullivan JDB , Nicu C , Picard M , et al. The biology of human hair greying. Biol Rev Camb Philos Soc. 2021;96(1):107‐128. 32965076 10.1111/brv.12648

[68] Nishimura EK , Granter SR , Fisher DE . Mechanisms of hair graying: incomplete melanocyte stem cell maintenance in the niche. Science. 2005;307(5710):720‐724. 15618488 10.1126/science.1099593

[69] Cinat D , Coppes RP , Barazzuol L . DNA damage‐induced inflammatory microenvironment and adult stem cell response. Front Cell Dev Biol. 2021;9:729136. 34692684 10.3389/fcell.2021.729136 PMC8531638

[70] Hasty P , Campisi J , Hoeijmakers J , van Steeg H , Vijg J . Aging and genome maintenance: lessons from the mouse? Science. 2003;299(5611):1355‐1359. 12610296 10.1126/science.1079161

[71] Inomata K , Aoto T , Binh NT , et al. Genotoxic stress abrogates renewal of melanocyte stem cells by triggering their differentiation. Cell. 2009;137(6):1088‐1099. 19524511 10.1016/j.cell.2009.03.037

[72] Kudlova N , Slavik H , Duskova P , et al. An efficient, non‐invasive approach for in‐vivo sampling of hair follicles: design and applications in monitoring DNA damage and aging. Aging. 2021;13(23):25004‐25024. 34874896 10.18632/aging.203744 PMC8714131

[73] Rachmin I , Lee JH , Zhang B , et al. Stress‐associated ectopic differentiation of melanocyte stem cells and ORS amelanotic melanocytes in an ex vivo human hair follicle model. Exp Dermatol. 2021;30(4):578‐587. 33598985 10.1111/exd.14309 PMC8600567

[74] Ezzedine K , Eleftheriadou V , Whitton M , van Geel N . Vitiligo. Lancet North Am Ed. 2015;386(9988):74‐84. 10.1016/S0140-6736(14)60763-7 25596811

[75] Le Poole IC , van den Wijngaard RM , Westerhof W , Dutrieux RP , Das PK . Presence or absence of melanocytes in vitiligo lesions: an immunohistochemical investigation. J Invest Dermatol. 1993;100(6):816‐822. 7684427 10.1111/1523-1747.ep12476645

[76] Staricco RG . Amelanotic melanocytes in the outer sheath of the human hair follicle. J Invest Dermatol. 1959;33:295‐297. 13833821 10.1038/jid.1959.154

[77] Meyer KC , Klatte JE , Dinh HV , et al. Evidence that the bulge region is a site of relative immune privilege in human hair follicles. Br J Dermatol. 2008;159(5):1077‐1085. 18795933 10.1111/j.1365-2133.2008.08818.x

[78] Ito T , Ito N , Saatoff M , et al. Maintenance of hair follicle immune privilege is linked to prevention of NK cell attack. J Invest Dermatol. 2008;128(5):1196‐1206. 18160967 10.1038/sj.jid.5701183

[79] Wu Y , Dai Y , Peng J , Xu A , Song X . Increased expression of beta2‐adrenoceptors is involved in vitiligo‐associated grey hair. J Eur Acad Dermatol Venereol. 2022;36(11):e949‐e951. 35770460 10.1111/jdv.18380

[80] Han X , Chang L , Qiu Z , et al. Micro‐injury induces hair regeneration and vitiligo repigmentation through Wnt/β‐catenin pathway. Stem Cells Dev. 2022;31(5‐6):111‐118. 35044224 10.1089/scd.2021.0276

[81] Lin X , Meng X , Lin J . The possible role of Wnt/beta‐catenin signalling in vitiligo treatment. J Eur Acad Dermatol Venereol. 2023;37(11):2208‐2221. 36912722 10.1111/jdv.19022

[82] Birlea SA , Costin GE , Roop DR , Norris DA . Trends in regenerative medicine: repigmentation in vitiligo through melanocyte stem cell mobilization. Med Res Rev. 2017;37(4):907‐935. 28029168 10.1002/med.21426 PMC5466503

[83] Murase D , Hachiya A , Amano Y , Ohuchi A , Kitahara T , Takema Y . The essential role of p53 in hyperpigmentation of the skin via regulation of paracrine melanogenic cytokine receptor signaling. J Biol Chem. 2009;284(7):4343‐4353. 19098008 10.1074/jbc.M805570200

[84] Minder EI , Barman‐Aksoezen J , Schneider‐Yin X . Pharmacokinetics and pharmacodynamics of afamelanotide and its clinical use in treating dermatologic disorders. Clin Pharmacokinet. 2017;56(8):815‐823. 28063031 10.1007/s40262-016-0501-5

[85] Toh JJH , Chuah SY , Jhingan A , Chong WS , Thng STG . Afamelanotide implants and narrow‐band ultraviolet B phototherapy for the treatment of nonsegmental vitiligo in Asians. J Am Acad Dermatol. 2020;82(6):1517‐1519. 31987791 10.1016/j.jaad.2020.01.035

[86] Lim HW , Grimes PE , Agbai O , et al. Afamelanotide and narrowband UV‐B phototherapy for the treatment of vitiligo: a randomized multicenter trial. JAMA Dermatol. 2015;151(1):42‐50. 25230094 10.1001/jamadermatol.2014.1875

[87] Vinay K , Dogra S , Parsad D , et al. Clinical and treatment characteristics determining therapeutic outcome in patients undergoing autologous non‐cultured outer root sheath hair follicle cell suspension for treatment of stable vitiligo. J Eur Acad Dermatol Venereol. 2015;29(1):31‐37. 10.1111/jdv.12426 24628828

[88] Mohanty S , Kumar A , Dhawan J , Sreenivas V , Gupta S . Noncultured extracted hair follicle outer root sheath cell suspension for transplantation in vitiligo. Br J Dermatol. 2011;164(6):1241‐1246. 21275943 10.1111/j.1365-2133.2011.10234.x

[89] Rodrigues M , Kosaric N , Bonham CA , Gurtner GC . Wound healing: a cellular perspective. Physiol Rev. 2019;99(1):665‐706. 30475656 10.1152/physrev.00067.2017 PMC6442927

[90] Morasso MI , Tomic‐Canic M . Epidermal stem cells: the cradle of epidermal determination, differentiation and wound healing. Biol Cell. 2005;97(3):173‐183. 15715523 10.1042/BC20040098 PMC1283090

[91] Chou WC , Takeo M , Rabbani P , et al. Direct migration of follicular melanocyte stem cells to the epidermis after wounding or UVB irradiation is dependent on Mc1r signaling. Nat Med. 2013;19(7):924‐929. 23749232 10.1038/nm.3194 PMC3859297

[92] Garcin CL , Ansell DM , Headon DJ , Paus R , Hardman MJ . Hair follicle bulge stem cells appear dispensable for the acute phase of wound re‐epithelialization. Stem Cells. 2016;34(5):1377‐1385. 26756547 10.1002/stem.2289 PMC4985639

[93] Garcia AM , McLaren CE . Melanoma: is hair the root of the problem? Pigment Cell Melanoma Res. 2011;24(1):110‐118. 20880199 10.1111/j.1755-148X.2010.00782.x PMC3768291

[94] Moon H , Donahue LR , Choi E , et al. Melanocyte stem cell activation and translocation initiate cutaneous melanoma in response to UV exposure. Cell Stem Cell. 2017;21(5):665‐678.e666. 29033353 10.1016/j.stem.2017.09.001 PMC9004284

[95] Harris ML , Pavan WJ . Postnatal lineage mapping of follicular melanocytes with the Tyr::CreER(T) (2) transgene. Pigment Cell Melanoma Res. 2013;26(2):269‐274. 23176440 10.1111/pcmr.12048 PMC4034131

[96] Bosenberg M , Muthusamy V , Curley DP , et al. Characterization of melanocyte‐specific inducible Cre recombinase transgenic mice. Genesis. 2006;44(5):262‐267. 16676322 10.1002/dvg.20205

[97] Sun Q , Lee W , Mohri Y , et al. A novel mouse model demonstrates that oncogenic melanocyte stem cells engender melanoma resembling human disease. Nat Commun. 2019;10(1):5023. 31685822 10.1038/s41467-019-12733-1 PMC6828673

[98] Köhler C , Nittner D , Rambow F , et al. Mouse cutaneous melanoma induced by mutant BRaf arises from expansion and dedifferentiation of mature pigmented melanocytes. Cell Stem Cell. 2017;21(5):679‐693.e676. 29033351 10.1016/j.stem.2017.08.003

[99] Viros A , Sanchez‐Laorden B , Pedersen M , et al. Ultraviolet radiation accelerates BRAF‐driven melanomagenesis by targeting TP53. Nature. 2014;511(7510):478‐482. 24919155 10.1038/nature13298 PMC4112218

[100] Hoerter JD , Bradley P , Casillas A , et al. Does melanoma begin in a melanocyte stem cell? J Skin Cancer. 2012;2012:571087. 23316368 10.1155/2012/571087 PMC3536063

[101] An L , Kim D , Donahue LR , et al. Sexual dimorphism in melanocyte stem cell behavior reveals combinational therapeutic strategies for cutaneous repigmentation. Nat Commun. 2024;15(1):796. 38280858 10.1038/s41467-024-45034-3 PMC10821900

[102] Potter SS . Single‐cell RNA sequencing for the study of development, physiology and disease. Nat Rev Nephrol. 2018;14(8):479‐492. 29789704 10.1038/s41581-018-0021-7 PMC6070143

[103] Jovic D , Liang X , Zeng H , Lin L , Xu F , Luo Y . Single‐cell RNA sequencing technologies and applications: a brief overview. Clin Transl Med. 2022;12(3):e694. 35352511 10.1002/ctm2.694 PMC8964935

[104] Zhang JZ , Termglinchan V , Shao NY , et al. A human iPSC double‐reporter system enables purification of cardiac lineage subpopulations with distinct function and drug response profiles. Cell Stem Cell. 2019;24(5):802‐811.e805. 30880024 10.1016/j.stem.2019.02.015 PMC6499654

[105] Hsu MN , Chang YH , Truong VA , Lai PL , Nguyen TKN , Hu YC . CRISPR technologies for stem cell engineering and regenerative medicine. Biotechnol Adv. 2019;37(8):107447. 31513841 10.1016/j.biotechadv.2019.107447

[106] Antao AM , Karapurkar JK , Lee DR , Kim KS , Ramakrishna S . Disease modeling and stem cell immunoengineering in regenerative medicine using CRISPR/Cas9 systems. Comput Struct Biotechnol J. 2020;18:3649‐3665. 33304462 10.1016/j.csbj.2020.11.026 PMC7710510

[107] Baggiolini A , Callahan SJ , Montal E , et al. Developmental chromatin programs determine oncogenic competence in melanoma. Science. 2021;373(6559):eabc1048. 34516843 10.1126/science.abc1048 PMC9440978

[108] Motter Catarino C , Cigaran Schuck D , Dechiario L , Karande P . Incorporation of hair follicles in 3D bioprinted models of human skin. Sci Adv. 2023;9(41):eadg0297. 37831765 10.1126/sciadv.adg0297 PMC10575578

[109] Mahla RS . Stem cells applications in regenerative medicine and disease therapeutics. Int J Cell Biol. 2016;2016:6940283. 27516776 10.1155/2016/6940283 PMC4969512

[110] Tang J , Fewings E , Chang D , et al. The genomic landscapes of individual melanocytes from human skin. Nature. 2020;586(7830):600‐605. 33029006 10.1038/s41586-020-2785-8 PMC7581540

